# Effects of a Protic Ionic Liquid on the Reaction Pathway during Non-Aqueous Sol–Gel Synthesis of Silica: A Raman Spectroscopic Investigation

**DOI:** 10.3390/ijms15046488

**Published:** 2014-04-16

**Authors:** Anna Martinelli

**Affiliations:** Department of Chemical and Biological Engineering, Chalmers University of Technology, Kemivägen 4, 41296 Göteborg, Sweden; E-Mail: anna.martinelli@chalmers.se; Tel.: +46-0-31-7723002

**Keywords:** ionic liquids, silica, ionogels, sol-gel, Raman spectroscopy

## Abstract

The reaction pathway during the formation of silica via a two-component “non-aqueou” sol-gel synthesis is studied by *in situ* time-resolved Raman spectroscopy. This synthetic route is followed with and without the addition of the protic ionic liquid 1-ethylimidazolium bis(trifluoromethanesulfonyl)imide (C_2_HImTFSI) in order to investigate its effect on the reaction pathway. We demonstrate that Raman spectroscopy is suitable to discriminate between different silica intermediates, which are produced and consumed at different rates with respect to the point of gelation. We find that half-way to gelation monomers and shorter chains are the most abundant silica species, while the formation of silica rings strongly correlates to the sol-to-gel transition. Thus, curling up of linear chains is here proposed as a plausible mechanism for the formation of small rings. These in turn act as nucleation sites for the condensation of larger rings and thus the formation of the open and polymeric silica network. We find that the protic ionic liquid does not change the reaction pathway *per se*, but accelerates the cyclization process, intermediated by the faster inclusion of monomeric species.

## 1. Introduction

Sol-gel synthesis is a very important chemical route to achieve silica based materials of relevance for many diverse applications [1,2]. The traditional pathway for SiO_2_ gel formation is the catalytic polycondensation of tetra-alkoxysilanes, e.g., TMOS or TEOS, in an alcoholic environment [3]. In contrast, in the much less common two-component *non-aqueous* sol-gel route the silica precursor reacts directly with a strong carboxylic acid, such as formic acid, that serves as the solvent and the catalyst at the same time [4]. Transparent monolithic gels can thus be obtained that have gelation times at least two orders of magnitude shorter than gels obtained through the conventional route, and physical properties that closely resemble those of conventional acid-catalyzed gels.

The attention for this alternative sol-gel route has recently boosted, since it is of great interest for the preparation of composites of silica and ionic liquids, also known as ionogels, which display high potential as materials for both catalytic and electrolytic applications [5–8]. Ionogels bring together the robustness of the silica network with the liquid-like dynamic properties of the ionic liquid, with the advantage of safety and non-volatility as opposed to conventional electrolytes based on organic solvents. Ionogel electrolytes of this kind have been obtained with different ionic liquids [5–14], mainly aprotic imidazolium such as C*_n_*C_1_ImTFSI [5,9,11,13,14] and only to a much lesser extent using ionic liquids based on a protic cationic structure [8,12]. The main focus so far has been the characterization of the chemico-physical properties of these materials as-prepared [8,10,11], while the mechanism of formation during the sol-gel reaction has, to the best of our knowledge, been investigated to a very limited extent. Suitable techniques to investigate *in situ* the sol-gel synthesis of silica include Raman and nuclear magnetic resonance (NMR) spectroscopies, which can selectively probe different silica intermediates [15,16], and high-brilliance synchrotron X-ray scattering [17] that in the small angle scattering setup can reveal information on the size and aggregation state of the silica particles forming the gel, in particular the fractal dimension of the silica network. In this context, we have recently studied the evolution of the reacting species by *in situ* Raman and NMR spectroscopy [9], as well as by simultaneous *μ*-Raman and *μ*-focused X-ray scattering [18]. However, these studies had at focus the behavior of the ionic liquid phase, while the mechanism of silica growth during the non-aqueous sol-gel route, and specifically in the presence of an ionic liquid, remains unexplored. Understanding this mechanism is of utmost importance since the competition of hydrolysis and condensation reactions that lead to gelation greatly influences the properties of the final material [19,20].

This knowledge gap is partially filled in by this paper, which demonstrates how time-resolved Raman spectroscopy can be used to follow sol-gel reactions *in situ* and discriminate between different silica intermediates. Both hydrolysis and condensation reactions are investigated, as well as the effect of including a protic ionic liquid on the reaction pathway. Differently from the case of traditional sol-gel reactions in which silica species are highly diluted in an aqueous solution, the two-component non-aqueous sol-gel route investigated here offers the advantage of high silica concentration and thereby a high selectivity for different species despite the short reaction times. In this study we emphasize the correlation between structural evolution and point of gelation, in order to achieve a deeper insight on the structural units that contribute to the rigidity of the gel. In particular, we analyze in detail the frequency evolution of the spectral feature at ~490 cm^−1^, and acquire information on the network -Si–O–Si- bond angles and on the size of the silica rings [21]. Along with revealing the effects of adding a protic ionic liquid in the sol-gel synthesis, this work also contributes to the ongoing debate whether the reaction pathway under acidic conditions and sub-stoichiometric water contents follows the model of linear chain growth [22,23], or that of cyclization reactions competing with chain extension [24,25], as also discussed by Depla *et al.* [26]. Finally, this work also aims to highlight the potential of Raman spectroscopy as a multi-faceted technique to characterize ionic liquid derived materials.

## 2. Results and Discussion

### 2.1. Assignment of Vibrational Modes

**Figure 1.**
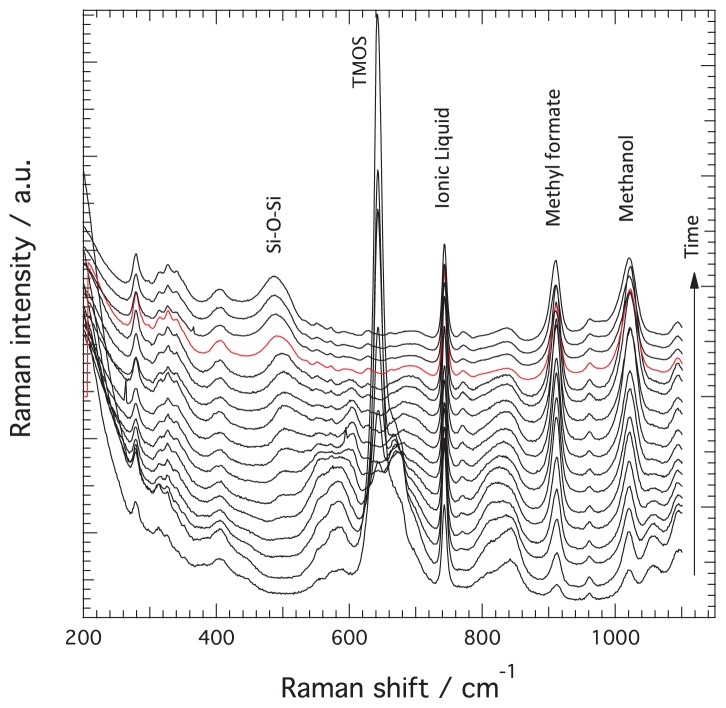
A selection of Raman spectra collected during the sol-gel reaction in the presence of the protic ionic liquid C_2_HImTFSI. Spectra recorded at increasing times (indicated by the vertical arrow) are shown from bottom to top with a vertical offset. The spectrum collected at the time of gelation is highlighted in red. Characteristic Raman frequencies associated to silica, TMOS, ionic liquid, methyl formate, and methanol are also indicated.

Figure 1 shows the evolution of the Raman spectra recorded at different times, from very early after mixing the reagents to well beyond the gelation point, see the arrow for the direction of time. In this Figure only spectra recorded for the reaction occurring in the presence of the ionic liquid are shown as a representative case. For clarity, spectra are shown with a vertical off-set and the spectrum recorded at the time of gelation, t*_gel_*, is highlighted in red. This selection of Raman spectra shows clear spectral changes with time. The Raman peaks that undergo major intensity changes are those at 910 and 1020 cm^−1^ assigned to methyl formate and methanol respectively, at 643 cm^−1^ assigned to the symmetric Si–OCH_3_ stretching in TMOS, and at ~490 cm^−1^ attributed to Si–*O*–Si bending modes in *n*-membered rings of the silica network. The intensity of the signature at ~740 cm^−1^, assigned to the expansion-contraction mode of the TFSI anion in the ionic liquid [27], does not change with time since the ionic liquid does not participate in the reaction, hence this Raman peak can be used as an internal standard to normalize Raman intensities. This procedure compensates for density fluctuations and dilution effects. The time evolution of these peaks has been extensively discussed in reference [9], revealing that the reactions that occur in solution can be summarized as:

(1) $$SiOCH_{3}+H_{2}O\to SiOH+HOCH_{3}$$

(2) $$SiOCH_{3}+HOOCH\to SiOH+CH_{3}OOCH$$

(3) $$SiOH+SiOCH_{3}\to SiOS_{i}+HOCH_{3}$$

(4) $$SiOH+SiOH\to SiOSi+H_{2}O$$

which result in the –OH substitution of TMOS, Equations (1) and (2), and in the formation of Si–*O*–Si bonds, Equations (3) and (4).

**Figure 2.**
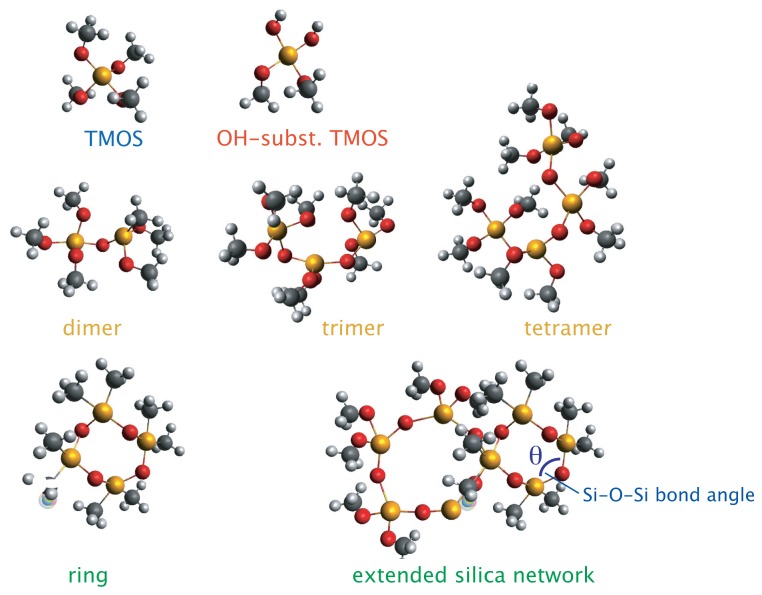
Different silica intermediates participating in the sol-gel reaction, *i.e.*, monomers (top), linear chains (middle), and cyclic forms or *n*-membered rings (bottom). Atom labels: Si (yellow), O (red), C (grey), and H (white). The inter-tetrahedral bond angle *θ* is also indicated.

In reference [9], however, the more subtle but very significant changes that occur in the regions 510–620, 640–720, and 780–880 cm^−1^, had not been inspected. The assignment of these vibrations has been been extensively reviewed in the works of Depla *et al.* [26], Mulder *et al.* [22], and Lippert *et al.* [28], in which the hydrolysis and condensation reactions during the formation of silica from TMOS or TEOS through classical sol-gel syntheses have been thoroughly investigated combining both experimental Raman measurements and theoretical calculations, with the support from ^29^Si NMR spectroscopy data [26]. As summarized in Table 1, the formation of linear Si–O–Si species, e.g., tetramer, trimer, or dimer, will give Raman vibrations in the 500–610 cm^−1^ range, partially hydrolyzed TMOS species, e.g., Si(OCH_3_)_2_(OH)_2_ or Si(OCH_3_)_1_(OH)_3_, will appear in the region 640–720 cm^−1^, while the vibrations in the range 780–880 cm^−1^ are assigned to Si–O symmetric stretching in intermediate species of different coordination states. Note that a signature at ~830 cm^−1^ is present also in the spectrum of TMOS, see Figure 2B in reference [9]. The feature at ~490 cm^−1^ is a signature for the formation of the silica network and of the presence of *n*-membered rings that can vary both in size and in Si–*O*–Si bond angles [21]. In Figure 2 the different silica intermediate species, *i.e.*, TMOS, –OH substituted TMOS, linear chains, and rings, are schematically shown to facilitate the discussion that follows.

### 2.2. Fast Hydrolysis Reactions

**Figure 3.**
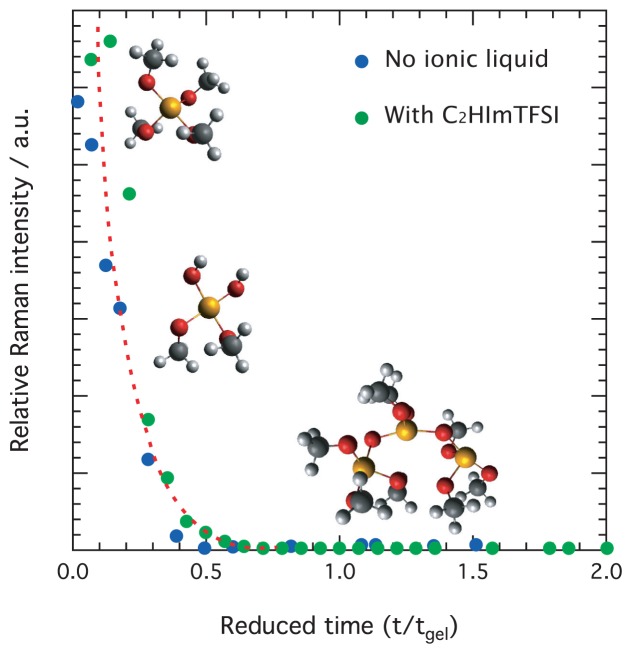
Time evolution of the Raman intensity associated to the symmetric Si–O–Si stretching in TMOS (644 cm^−1^) for the solutions containing no ionic liquid and 0.05 mole ratio towards TMOS of C_2_HImTFSI. A reduced time scale is used in the *x*-axis.

**Figure 4.**
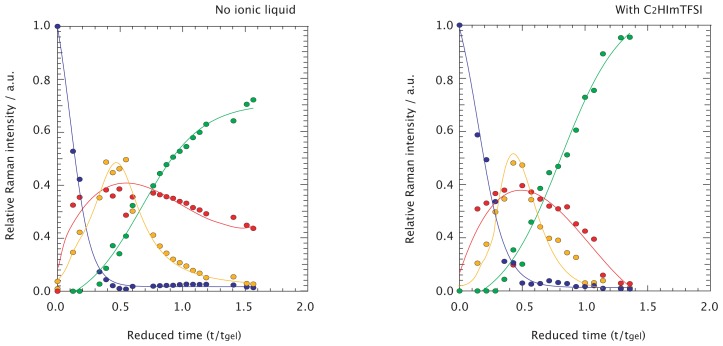
Relative Raman contributions to the spectral range 400–800 cm^−1^ from OH-substituted TMOS (blue), monomeric (red), linear (yellow), and cyclic (green) species, for no ionic liquid (**left**) and for the C_2_HImTFSi ionic liquid added (**right**) solutions.

Figure 3 shows the rate of consumption of TMOS on a reduced time scale, that is as a function of t/t*_gel_* where t/t*_gel_* = 1 corresponds to the time of gelation. Here it is worth recalling the strong effect on t*_gel_* of the ionic liquid addition [9], and that in this study t*_gel_* for for no ionic liquid added is 170 min while t*_gel_* for the ionogel with *x* = 0.05 of C_2_HImTFSI is 65 min. Data collected from solutions with and without the ionic liquid are shown in the same plot. From this figure it can be appreciated that the consumption of TMOS is very fast, and appears almost complete at t*_gel_* = 0.5. Here, it is crucial to remark that since the vibration at 643 cm^−1^ arises from the symmetric stretching of the four Si–O bonds in Si–(OCH_3_)_4_, even the reaction of one –OCH_3_ group only would result in symmetry breaking and thus in a decrease of the 643 cm^−1^ intensity. Hence, a ~zero intensity of the Raman peak at 643 cm^−1^ does not necessarily mean complete hydrolysis, rather the –OH substitution of at least one functional group for all TMOS molecules. In fact, the evolution of ^1^H NMR intensities recorded during an equivalent sol-gel reaction to that investigated in this work indicates that at t/t*_gel_* = 0.5 approximately 20% of the –OCH_3_ groups are not reacted (see Figure 4B in reference [9]). Nevertheless, the same work also shows that TMOS is completely hydrolyzed at t/t*_gel_* = 1. Very fast hydrolysis reactions, with respect to the time of gelation, are typical of sol-gel reactions that occur in acidic conditions and at sub-stoichiometric water contents [26,28]. These conditions are in fact reproduced in the systems investigated here, due to the very high formic acid concentration and very low water content in the initial solution. More precisely, considering that formic acid is commercially available with 4 wt % of water, our initial solution actually contains 0.3 water molecules per TMOS unit, which corresponds to a H_2_O:TMOS ratio of *r* = 0.3. This is much smaller than the values of 1–25 normally used in the traditional sol-gel synthesis of silica [19]. In addition, the data shown in Figure 3 reveal that the rate at which TMOS is consumed is very little affected when the reaction occurs in the presence of the protic ionic liquid C_2_HImTFSI.

### 2.3. Competing Condensation Reactions

In Figure 4 the relative integrated Raman intensities of different silica species, *i.e.*, monomeric, linear, and cyclic, are plotted as a function of reduced time, t/t*_gel_*, together with the time evolution of the Raman feature assigned to TMOS. As previously found from both experimental and theoretical works [22,26,28], the Raman signatures of linearly polymerized silica (tetramers, trimers, and dimers) are very close in frequency and may slightly shift as a consequence of different configurations or degree of –OH substitution. Significant peak overlap could than make a quantitative analysis of the individual signatures of ambiguous interpretation. Therefore, as a first approximation, we find it more appropriate to consider these signatures altogether, the sum of the dimer, trimer, and tetramer intensities being representative of the *linear* silica species:

(5) $$I_{linear}=\sum I_{dim}+I_{trim}+I_{tetram}=\sum I_{604}+I_{581}+I_{554}$$

where the subscripts on the right-hand term indicate the approximate frequency at which the Raman vibration of that silica species is found. Similarly, the TMOS molecules with different degrees of –OH substitution (*i.e.*, Si(OH)*_i_*(OCH_3_)_4−_*_i_*, where *i* can vary from 1 to 4) altogether represent the *monomeric* species:

(6) $$I_{monomer}=\sum I_{Si{\left(OH\right)}_{i}{\left(OCH3\right)}_{4-i}}=\sum I_{674}+I_{696}+I_{726}(+I_{795})$$

where any species with a value of *i* larger than one represents a monomer available for further condensation reactions. The successive increase in Raman frequency with substitution of the –OCH_3_ groups by –OH is consistent with the smaller reduced mass of the vibrating unit. Nevertheless, the assignment of the vibrational mode at 795 cm^−1^ is more uncertain, and has been assigned to silica dimers by Artaki *et al.* [31] and to silicic acid or Si(OH)_4_ by Lippert *et al.* [28]. In this work we do not observe a shoulder at 795 cm^−1^ but at 810 cm^−1^, which in our opinion can not be assigned to silicic acid since the shift from 726 to 810 cm^−1^ is too large to be motivated by mass reduction from only one extra –OH group. On the other hand, the assignment to dimers is in contrast with the assignment scheme discussed above. Moreover, as clearly shown in the Raman spectra plotted in Figure 1, the shoulder at ~810 cm^−1^ grows and diminishes consistently with the features in the range 510–620 cm^−1^, assigned to linear silica species. We therefore propose the assignment of the 810 cm^−1^ mode to Si–O(CH_3_) vibrations in side-groups of central parts of trimers or tetramers, see also Table 1. As a consequence, in this analysis we consider only the signatures in the range 670–730 cm^−1^ as representative of the monomeric species, while the term in parenthesis in Equation (6) is not taken into account when calculating relative Raman contributions. The broad signature growing in the spectral range below 530 cm^−1^ is attributed to Si–O–Si bending modes in *n*-membered rings with partial contribution from the two adjacent Si atoms [21]. In analogy to the approaches above, rings of different nature are considered altogether to represent the *cyclic* species:

(7) $$I_{ring}=\sum I_{430-530}$$

Hence, in Figure 4 the relative Raman intensities so calculated represent relative contributions to the entire 450–750 cm^−1^ spectral range. To clarify, the linear species shown in yellow are in fact *I_linear_*/(*I_TMOS_* + *I_monomer_* + *I_linear_* + *I_ring_*). The reader should consider, however, that differently from NMR spectroscopy were NMR intensities are directly proportional to concentrations, in Raman spectroscopy the Raman intensity is also proportional to the Raman scattering cross section, which can variate between molecular groups. This implies that the conversion of relative Raman intensities into relative population is not straightforward. However, the qualitative trends of produced and consumed species as shown in Figure 4 remains sound. As shown in Figure 4A for no ionic liquid added to the reaction, the species most readily formed are partially –OH substituted TMOS, *i.e.*, Si(OH)_2_(OCH_3_)_2_, Si(OH)_3_(OCH_3_)_1_ *etc.* shown in red, followed with a little delay by the linearly polymerized silica species, shown in yellow. Although this Figure confirms a very fast hydrolysis reaction, differently from the case of traditional acid-catalyzed sol-gel synthesis of silica [19] where complete hydrolysis precedes condensation we here observe an earlier formation of monomeric and linear species. The population of both these species increases with time, up to approximately half-way to the gelation point, t/t*_gel_* = 0.5. This is also the time at which the population of linear species starts to dramatically decrease while that of silica rings (green) starts to raise. As can be deduced from intensity changes in the time scale t/t*_gel_* = 0.5–1.0, silica rings are formed mainly at the expenses of linear species, which is also consistent with the delay with which the cyclic forms are produced. After gelation, the cyclic species dominate the spectral range investigated, with a non-negligible contribution from monomers.

**Figure 5.**
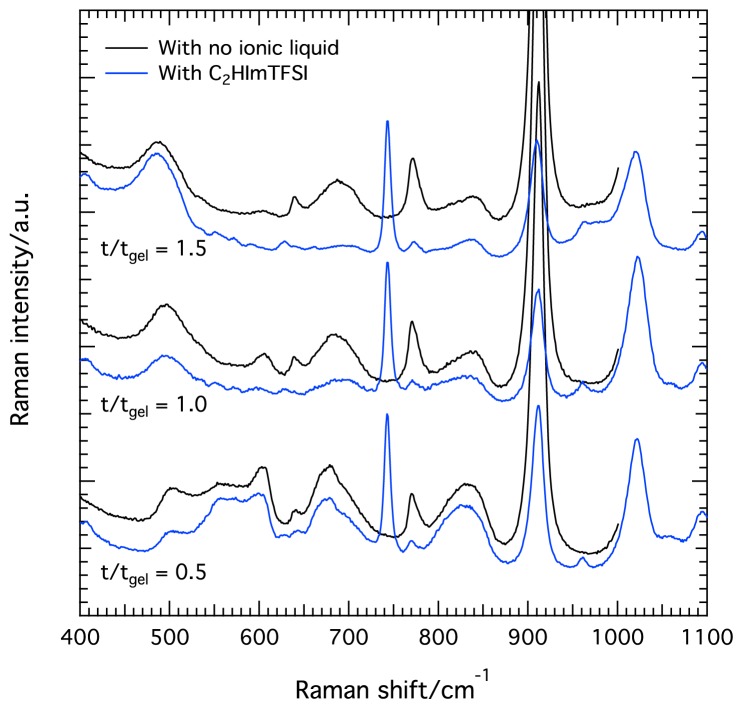
Raman spectra recorded in solutions/gels with (blue) and without (black) the ionic liquid C_2_HImTFSi at different reaction times, *i.e*., at t/t*_gel_* = 0.5, 1.0, and 1.5.

The intensity evolution with time of the different silica species is essentially the same in the presence of the ionic liquid C_2_HImTFSI, as shown in Figures 4B. One clear difference, however, is that after t*_gel_* the consumption of monomers and the increase of ring population is much faster than for the case of no ionic liquid added. At t/t*_gel_* = 1.5 practically no monomers or oligomers are present in the ionogel and the spectrum is dominated by the feature peaked at ~490 cm^−1^. These differences suggest that the cyclization process is significantly accelerated in the presence of the ionic liquid. The different kinetics can be visualized in Figure 5, in which Raman spectra recorded at comparable t/t*_gel_* times are plotted for solutions with and without the ionic liquid C_2_HImTFSI.

**Figure 6.**
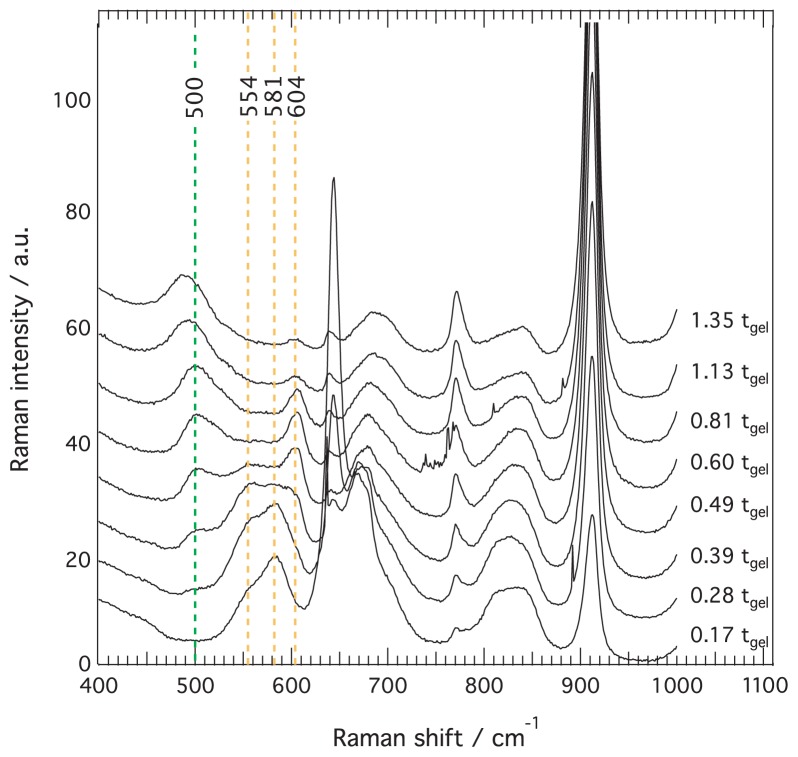
Close-up of the spectral range 400–1000 cm^−1^ showing spectra recorded in the time domain 0.17–1.35 t/t*_gel_*, for the solution with no ionic liquid added. Spectral signatures assigned to cyclic (green) and linear (yellow) species are indicated by dashed lines.

The condensation mechanism has been further analyzed by a closer inspection of the Raman spectral region 400–1000 cm^−1^, with focus on spectra recorded close to the gelation point, *i.e.*, between 0.17 and 1.35 t/t*_gel_*, see Figure 6. The dotted lines indicate the distinct features at ~554, ~581, and ~604 cm^−1^, that increase and decrease at different rates. According to the works of Mulder *et al.* [22] and Depla *et al.* [26] and as summarized in Table 1, these are assigned to non-OH substituted tetra-, tri-, and dimers of silica respectively. From Figure 6 it is clear that after a concomitant increase of both trimers and tetramers, at ~ 0.39 t/t*_gel_* these start to be consumed with an apparently faster consumption of the trimers (581 cm^−1^) as compared to the tetramers (554 cm^−1^). This consumption is concomitant with the formation of cyclic silica forms (feature at ~490 cm^−1^), while the feature at ~604 cm^−1^ remains of significant intensity, see spectra between 0.49 and 0.81 t/t*_gel_*. However, from 1.13 t/t*_gel_* also this feature shows an intensity decrease, while the intensity of the signal at ~490 cm^−1^ is observed to further and smoothly increase. The observation of the ~604 cm^−1^ feature at later times than that at ~581 or ~554 cm^−1^, implies that dimers should exist in solution together with rings. These species would then act as cross linkers between rings, a process that seems to coincide with the set in of gelation. This scenario, however, is in contrast with other time-resolved studies on the polymerization of TEOS, which have shown that the consumption of dimers precedes the formation of trimers or tetramers [22–24]. However, as discussed by Depla *et al.* [26] this feature has contributions from end-groups of chains (602 cm^−1^), wherefore for consistency with previous reaction models the residual peak at ~604 cm^−1^ may be more correctly attributed to non-condensed Si–OCH_3_ side groups of small silica rings. This assignment is supported by that the decrease of the ~604 cm^−1^ feature is concomitant with the increased degree of cyclic condensation, *vide infra*.

### 2.4. Evolution of the Cyclic Forms of Silica

As extensively discussed by Hehlen [21], the Raman feature at ~490 cm^−1^ is characteristic of all amorphous silica-derived materials, although its frequency can vary depending on the inter-tetrahedral Si–O–Si bond angles formed, and therefore on the size of the *n*-membered rings that build up the silica network. In his work, Hehlen concludes that by using a silica sample with known structure as a reference, the average inter-tetrahedral bond angles of an unknown silicate can be deduced from the reduced Raman spectrum in the frequency range of the ring breathing modes. Here, the reduced Raman spectrum G(*ω*) is related to the experimentally recorded Raman spectrum I(*ω*) through G(*ω*) ∝ I(*ω*)/(*ω*·[n(*ω*) + 1)], where *ω* is the Raman frequency (in cm^−1^) and n(*ω*) is the Bose occupation factor. We aim to use this finding to get new insights on the cyclization process during the sol-to-gel transition investigated.

**Figure 7.**
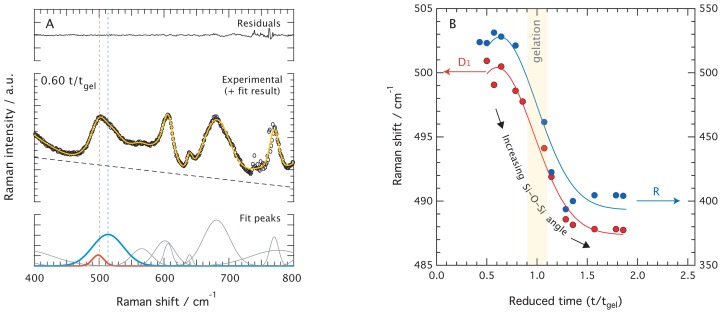
**Left:** Example of a peak-fit analysis for the spectrum of no ionic liquid added solution recorded at t/t*_gel_* = 0.60. The residuals (top), the fitting results (center), and the individual fitting components (bottom) are shown in separate panels. **Right:** Frequency evolution as a function of reduced time of the fitting components associated to the *n*-membered ring vibrations for the representative case of the ionic liquid added solution (same color coding as in left).

The peak fitting analysis of reduced Raman spectra reveals that the ~490 cm^−1^ feature must be deconvoluted into two components. In fact, as can be seen in Figure 7A for the representative case of the ionic liquid added gel at t/t*_gel_* = 0.60, this feature is broad and asymmetric. We also find that both these components systematically shift towards lower frequencies as the reaction proceeds, see also Figure S1 for further details on this evolution. The frequency shift of the two fitting components as a function of reduced time is displayed in Figure 7B, revealing that the broader component shifts from ~528 to ~408 cm^−1^ (blue), while the weaker one from ~501 to ~487 cm^−1^ (red). Judging from the frequency ranges in which these features variate, and based on many Raman spectroscopic studies on densified amorphous silica gels and glasses that display the characteristic broad feature at ~432 cm^−1^ and the so-called D_1_ defect mode at ~490 cm^−1^, these features are assigned to the Si–O–Si bending modes in non-planar network *n*-membered rings and the Si–O–Si in-phase “breathing mode” in planar 4-membered rings respectively [21]. While the“breathing mode” (D_1_) of the smaller rings do not involve a significant contribution from the two adjacent silicon atoms of the Si–O–Si bond, the vibrations that lead to the network modes (R) do so and are probably closer to pure bendings.

The relation proposed by Hehlen [21] between the Raman frequency shift for the Si–O–Si bending mode and the corresponding Si–O–Si bond angle is cos*θ*/2 = 7.323 · 10^−4^ · *ω*, where *ω* is the Raman frequency shift in cm^−1^. This relation has been established considering that the angle at the maximum of the distribution in v-SiO_2_ is 145° [21]. Using this, the frequency variation between ~501 and 487 cm^−1^ translates into Si–O–Si bond angles that vary from ~137.1° to ~138.2° in the time domain 0.7–2·t/t*_gel_*. On the other hand, the frequency change of the broader feature from ~528 to ~408 cm^−1^ in the same time domain corresponds to Si–O–Si angles varying from ~134.7° to ~146.1°, see Figure 7B. Although these values have been calculated for a gel material under structural evolution, they are in very good agreement with the results obtained for the D_1_ defect mode and the R network modes in densified silica [21]. The small bond angle variation observed for the D_1_ mode with reaction time is ascribed to a relaxation of the structure, while the more significant changes found for the R network mode are indicative of the formation of new and progressively larger rings. It is noteworthy that the major changes occur at around gelation, at t/t*_gel_* = 1, whereas at roughly t/t*_gel_* = 2 the frequency shift of both the D_1_ and the R network vibrational modes have reached a plateau indicating that no further structural changes take place. These findings indicate that while the first cyclic forms are 4-membered rings, after gelation (more exactly after t/t*_gel_* = 1.5) these contain on average 6 Si atoms. This is consistent with the results found for densified silica [21] where network frequencies at around 420 cm^−1^ are representative of Si–O–Si bond angles of about 144° and ≃6-membered rings, which is also the most probable silica ring size found by numerical simulations [32].

To summarize, the concomitant decrease of the component at ~560 cm^−1^ and the increase of a new one at below ~530 cm^−1^ reveals a smooth structural transition from linear tetramers, in the open or closed conformation [26], to 4-membered rings. The progressively more open and extended network is then formed by either ring-ring or ring-chain condensation reactions. This scenario finds support from our observation that oligomers longer than tetramers have not been detected, which is also in good agreement with the findings of Depla *et al.* who have investigated the formation of silica through an acid-catalyzed sol-gel reaction at very low water contents by NMR and Raman spectroscopy [26]. Since both the intensity and the frequency evolutions that we have investigated in the silica sensitive range 400–800 cm^−1^ are very similar on the time scale that extends to well beyond gelation with and without the ionic liquid, we conclude that the reaction pathway *per se* does not significantly change upon the addition of C_2_HImTFSI. In both cases the formation of *n*-membered rings is strongly correlated to the point of gelation, but we also observe that the cyclization process is much faster when C_2_HImTFSI is included in the reaction (see Section 2.3). The faster cyclization process can be in line with the role of ionic liquids as reaction catalysts as recently proposed by Karout *et al.* [33] as well as with our observation that small amounts of an imidazolium ionic liquid can slightly enhance the Q^4^/Q^3^ ratio in ionogels [34].

**Figure 8.**
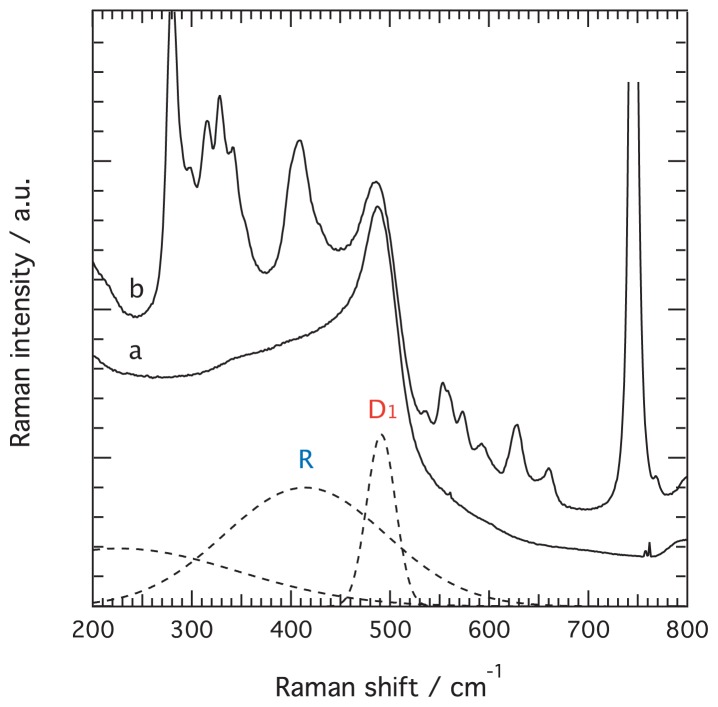
Raman spectra recorded on the aged gel (a) and aged ionogel (b), *i.e.*, six months after synthesis. The fitting components are shown as dashed lines.

That no significant structural changes occur after gelation is further confirmed by Raman spectra recorded for aged gels, *i.e.*, six months after preparation, which show that the Raman feature representing the silica network has still an apparent maximum at ~491 cm^−1^, and that the broader feature is still located at ~410–430 cm^−1^, see Figure 8. In these spectra no residual peaks are found in the range 520–780 cm^−1^, meaning that all silica intermediates have undergone condensation. The weak features in the spectrum of the aged ionogel between 550 and 700 cm^−1^ are intrinsic vibrations of the ionic liquid’s cation. The Raman spectra of both the aged gel and the aged ionogel also show a relatively strong signature at 977 cm^−1^, which is attributed to Si–OH stretching and thus indicates the presence of silanol groups, see Figure S2. The spectral features of most silica gels and glasses do show the presence of silanol groups, which however undergo condensation upon treatment at very high temperatures [35]. However, since we have recently shown that in ionogels the ionic liquid strongly interacts with the silica surface, more precisely with the aromatic head of *n*-alkyl-imidazolium ionic liquids oriented flatly to the silica surface forming a “bound and immobil” solvation layer [11], the surface chemistry in gels and ionogels may differ and result in different responses to heat treatment/dehydration. This aspect should be of importance when designing ionogels for real applications.

## 3. Experimental Section

### 3.1. Materials

The gels were prepared following the *non-aqueous* sol-gel route described in more detail elsewhere [9]. The ionic liquid 1-ethylimidazolium bis(trifluoromethanesulfonyl)imide (C_2_HImTFSI) was purchased from Iolitec (Hellbronn, Germany) and kept in an Ar-gas filled glovebox prior to use. The reactants tetramethyl orthosilicate (TMOS, 99% purity, Sigma-Aldrich, Stockholm, Sweden) and formic acid (FA, commercially available as 96% by weight in water, Sigma-Aldrich, Stockholm, Sweden) were mixed under vigorous stirring at a FA:TMOS molar ratio of 3:1, this being enough for the complete reaction of the Si(OCH_3_)_4_ groups in TMOS, as also reported in reference [9]. For the preparation of ionogels, the ionic liquid C_2_HImTFSI was immediately added to the TMOS/formic acid mixture at a molar ratio, *x*, with respect to TMOS, equal to 0.05, resulting in a transparent and homogeneous solution. The sol-gel reaction was let to occur into 2 mL vials, suitable for the *in situ* and time-resolved Raman spectroscopic measurements. In traditional sol-gel reactions the precursor particles e.g., TMOS or TEOS) present in solution undergo hydrolysis and condensation to result in dispersed silica nano-particles that aggregate to form a three-dimensional solid network. This solid network interpenetrated by the liquid phase (traditionally water) is what is referred to as a gel. The size of the aggregating particles, their surface roughness, and their degree of polymerization strongly depend on the synthesis conditions such as pH of the solution, as well as relative water and alcohol concentrations [36]. These reaction conditions in turn also influence the micro-structure of the final gel [20]. In the non-aqueous sol-gel route investigated here, gels and ionogels form under acidic conditions and sub-stoichiometric water contents, while in the ionogels the liquid phase that percolates the silica network is mainly the ionic liquid (as opposed to water).

### 3.2. Raman Spectroscopy

Raman spectra were recorded on an InVia Reflex Renishaw spectrometer (purchased from K-Analys AB, Uppsala, Sweden, the local sale contact for Renishaw plc, New Mills, UK) using the 785 nm laser wavelength (~300 mW of power), a 1200 L/mm grating, a peltier cooled CCD detector, and a x50 LWD Leica objective. The spectrometer was calibrated to the 1st order band at ~520 cm^−1^ of a Si wafer. Raman spectra were collected with the 180° backscattering collection geometry, with the monochromatic laser approaching the vials containing the reagents from above. All spectra were collected at room temperature at regular time intervals, of 5 or 10 min, while the focal point of the laser was manually adjusted before each measurement to ensure its location well inside the solution. For quantitative analyses, Raman spectra in the frequency range 400–1000 cm^−1^ were deconvoluted using Lorentzian functions and a linear background, whereby relative intensities were calculated as integrated areas under the peaks.

## 4. Conclusions

In this study we have followed the reaction pathway during the formation of silica via a non-aqueous sol-gel synthesis with and without the ionic liquid C_2_HImTFSI. In the latter case, the synthesis results in a so-called ionogel, a material that has recently raised significant interest for potential use in catalytic and electrolytic applications. By use of *in-situ* and time-resolved Raman spectroscopy we have been able to discriminate different silica species, from very early after mixing the reagents to well beyond gelation. We find that both with and without the protic ionic liquid, the hydrolysis of TMOS is very fast, and that up to ~t/t*_gel_* = 0.5 monomers and linear silica species are the most abundant. Only after ~t/t*_gel_* = 0.5 the cyclic species start to form, that possibly result from curling up of tetramers and progressively grow in both size and population. Noteworthy, the formation of *n*-membered silica rings is found to strongly correlate with the point of gelation, both with and without the ionic liquid. Thus, the protic ionic liquid C_2_HImTFSI does not change the reaction pathway *per se* but significantly accelerates the cyclization process, which seems to occur by faster incorporation of monomers into the silica network. These findings, that result from employing a detailed peak-fitting analysis on time-resolved Raman spectra, contribute to the field of sol-gel chemistry with new insights on the growth mechanism of silica under acidic and sub-stoichiometric water conditions. In particular, our results support the models where the cyclization reactions have an important contribution to structural growth and gelation.

## Supplementary Information



## Figures and Tables

**Table 1.** Raman frequency shifts (in cm^−1^) of different molecules and intermediates participating in the sol-gel reaction, together with reported or proposed assignments.

Molecule	Freq. [cm^−1^]	Assignment	Ref.
TMOS	644	*ν_s_* Si–O	[9]
TMOS	845	*ν_as_* Si–O	[9]
methyl formate	910	*ν* O–CH_3_	[9]
ionic Liquid	740	exp.-contr. of TFSI	[9,27]
methanol	1020	*ν* C–O	[9]
dimer	604	*ν* Si–O(Si) in Si_2_O(OCH_3_)_6_^a^	[22,26,28]
trimer	581	*ν* Si–O(Si) in Si_3_O_2_(OCH_3_)_8_	[22,26,28]
tetramer	554	*ν* Si–O(Si) in Si_4_O_3_(OCH_3_)_10_	[22,26,28]
*4*-membered rings (planar)	490	*δ* Si–O–Si b	[21]
*n*-membered rings (non-planar)	422–486	*δ* Si–O–Si c	[21]
OH-subst. TMOS	674	*ν* Si–O(H) in Si(OCH_3_)_3_OH	[28]
OH-subst. TMOS	696	*ν* Si–O(H) in Si(OCH_3_)_2_OH_2_	[28]
OH-subst. TMOS	726	*ν* Si–O(H) in Si(OCH_3_)_1_OH_3_	[28]
linear species	810	*ν* Si–O(CH_3_)	n.a.

a With contributions from chain end-groups [26];

b Pure ring “breathing modes” [29];

c With weak contributions from the adjacent Si atoms to the *O*–Si–*O* bond [30].

## References

[1] Ciriminna R., Fidalgo A., Pandarus V., Belard F., Ilharco L.M., Pagliaro M. (2013). The sol–gel route to advanced silica-based materials and recent applications. Chem. Rev.

[2] Kato M., Sakai-Kato K., Toyo’oka T. (2005). Silica sol–gel monolithic materials and their use in a variety of applications. J. Sep. Sci.

[3] Brinker C.J., Keefer K.D., Schaefer D.W., Assink R.A., Kay B.D., Ashley C.S. (1984). Sol–gel transition in simple silicates II. J. Non-Cryst. Solids.

[4] Sharp K.G. (1994). A two-component, non-aqueous route to silica gel. J. Sol-Gel Sci. Technol.

[5] Neouze M.-A., Le Bideau J., Gaveau P., Bellayer S., Vioux A. (2006). Ionogels, new materials arising from the confinement of ionic liquids within silica-derived networks. Chem. Mater.

[6] Le Bideau J., Viau L., Vioux A. (2011). Ionogels, ionic liquid based hybrid materials. Chem. Soc. Rev.

[7] Mutin P.H., Vioux A. (2013). Recent advances in the synthesis of inorganic materials via non-hydrolytic condensation and related low-temperature routes. J. Mater. Chem. A.

[8] Xie Z.-L., Xu H-B., Geßner A., Kumke M.U., Priebe M., Frommc K.M., Taubert A. (2012). A transparent, flexible, ion conductive, and luminescent PMMA ionogel based on a Pt/Eu bimetallic complex and the ionic liquid [Bmim][N(Tf)_2_]. J. Mater. Chem.

[9] Martinelli A., Nordstierna L. (2012). An investigation of the sol–gel process in ionic liquid-silica gels by time resolved Raman and ^1^H NMR spectroscopy. Phys. Chem. Chem. Phys.

[10] Wu C.-M., Lin S.-Y., Chen H.-L. (2012). Structure of a monolithic silica aerogel prepared from a short-chain ionic liquid. Microporous Mesoporous Mater.

[11] Nayeri M., Aronson M., Bernin D., Chmelka B.F., Martinelli A. (2014). Surface effects on the structure and mobility of the ionic liquid C_6_C_1_ImTFSI in silica gels. Soft Matter.

[12] Mizumo T., Watanabe T., Ohno H. (2008). Thermally stable and proton conductive ionogel based on brønsted acidic ionic liquid with the support of silicate network. Polym. J.

[13] Feng Y., Li H., Gan Q., Wang Y., Liu B., Zhang H. (2010). A transparent and luminescent ionogel based on organosilica and ionic liquid coordinating to Eu3+ ions. J. Mater. Chem.

[14] Horowitz A.I., Panzer M.J. (2012). High-performance, mechanically compliant silica-based ionogels for electrical energy storage applications. J. Mater. Chem.

[15] Fyfe C.A., Aroca P.A. (1997). Quantitative kinetic analysis by high-resolution ^29^Si NMR spectroscopy of the initial stages in the sol–gel formation of silica gel from tetraethoxysilane. Chem. Mater.

[16] Panitz J.-C., Wokaun A. (1995). Characterization of the sol–gel process using Raman spectroscopy. Organically modified silica gels prepared via the formic acid-alkoxide route. J. Sol-Gel Sci. Technol.

[17] Michaux F., Baccile N., Imperor-Clerc M., Malfatti L., Folliet N., Gervais C., Manet S., Meneau F., Pedersen J.S., Babonneau F. (2012). *In situ* time-resolved SAXS study of the formation of mesostructured organically modifeid silica thorugh modeling of micelles evolution during surfactant-templated self-assembly. Langmuir.

[18] Nayeri M., Karlsson M., Nygård K., Burghammer M., Reynolds M., Martinelli A. (2014). Nano-structural evolution of an imidazolium ionic liquid during sol-gel synthesis of silica. Langmuir.

[19] Brinker C.J. (1988). Hydrolysis and condensation of silicates: Effects on structure. J. Non-Cryst. Solids.

[20] Perullini M., Jobbagy M., Bilmes S.A., Torriani I.L., Candal V. (2011). Effect of synthesis conditions on the microstructure of TEOS derived silica hydrogels synthesized by the alcohol-free sol–gel route. J. Sol-Gel Sci. Technol.

[21] Hehlen B. (2010). Inter-tetrahedral bond angle of permanently densified silicas extracted from their Raman spectra. J. Phys. Condensed Matter.

[22] Mulder C.A.M., Damen A.A.J.M. (1987). Raman analysis of the initial stages of the hydrolysis and polymerization of tetraethylorthosilicate. J. Non-Cryst. Solids.

[23] Gnado J., Dhamelincourt P., Pelegris C., Traisnel M., Le Maguer Mayot A. (1996). Raman spectra of oligomeric species obtained by tetraethoxysilane hydrolysis-polycondensation process. J. Non-Cryst. Solids.

[24] Brus J., Karhan J., Kotlik P. (1996). ^29^Si NMR study of distribution of oligomers in polycondensation of tetraethoxysilane. Collect. Czechoslov. Chem. Commun.

[25] Bailey J.K., Macosko C.W., Mecartney M.L. (1990). Modeling the gelation of silicon alkoxides. J. Non-Cryst. Solids.

[26] Depla A., Lesthaeghe D., van Erp T.S., Aerts A., Houthoofd K., Fan F., Li C., van Speybroeck V., Waroquier M., Kirschhock C.E.A. (2011). ^29^Si NMR and UV-Raman investigation of initial oligomerization reaction pathways in acid-catalyzed silica sol-gel chemistry. J. Phys. Chem. C.

[27] Martinelli A., Matic A., Johansson P., Jacobsson P., Börjesson L., Fernicola A., Panero S., Scrosati B., Ohno H. (2011). Conformational evolution of TFSI^−^ in protic and aprotic ionic liquids. J. Raman Spectrosc.

[28] Lippert J.L., Melpolder S.B., Kelts L.M. (1988). Raman spectroscopic determination of the pH dependence of intermediates in sol-gel silicate formation. J. Non-Cryst. Solids.

[29] Pasquarello A., Car R. (1998). Identification of raman defect lines as signatures of ring structures in vitreous silica. Phys. Rev. Lett.

[30] Galeener F.L., Geissberger A.E. (1983). Vibrational dynamics in ^30^Si-substituted vitreous SiO_2_. Phys. Rev. B.

[31] Artaki I., Bradley M., Zerda T.W., Jonas J. (1985). NMR and Raman study of the hydrolysis reaction in sol-gel processes. J. Phys. Chem.

[32] Giacomazzi L., Umari P., Pasquarello A. (2009). Medium-range structure of vitreous SiO_2_ obtained through first-principles investigation of vibrational spectra. Phys. Rev. B.

[33] Karout A., Pierre A.C. (2009). Silica gelation catalysis by ionic liquids. Catal. Commun.

[34] Nayeri M. (2014). Department of Chemical and Biological Engineering.

[35] Björnström J., Martinelli A., Johnson J.R.T., Matic A., Panas I. (2003). Signatures of a drying SiO_2_·(H_2_O)*_x_* gel from Raman spectroscopy and quantum chemistry. Chem. Phys. Lett.

[36] Iler R.K. (1979). The Chemistry of Silica - Solubility, Polymerization, Colloid and Surface Properties, and Biochemistry.

