# Supplementary material for: Effects of a Protic Ionic Liquid on the Reaction Pathway during Non-Aqueous Sol–Gel Synthesis of Silica: A Raman Spectroscopic Investigation
Source: Int J Mol Sci. 2014 Apr 16;15(4):6488–503. doi: 10.3390/ijms15046488 (PMC4013642; doi:10.3390/ijms15046488)
Supplement: Supplementary file 1 [file ijms-15-06488-s001.pdf]

Supplementary Information

Figure S1. Experimentally recorded Raman spectra during the sol-gel reaction in the absence of the protic ionic liquid, for reaction times equal to 0.49, 0.60, 1.13, and 1.35 t/t_{gel}, from top to bottom. For each plot the fitting components, the fit results, and the fit residuals are shown. The red and blue curves found below 530 cm^{-1} are assigned to the D_1 and R network modes.

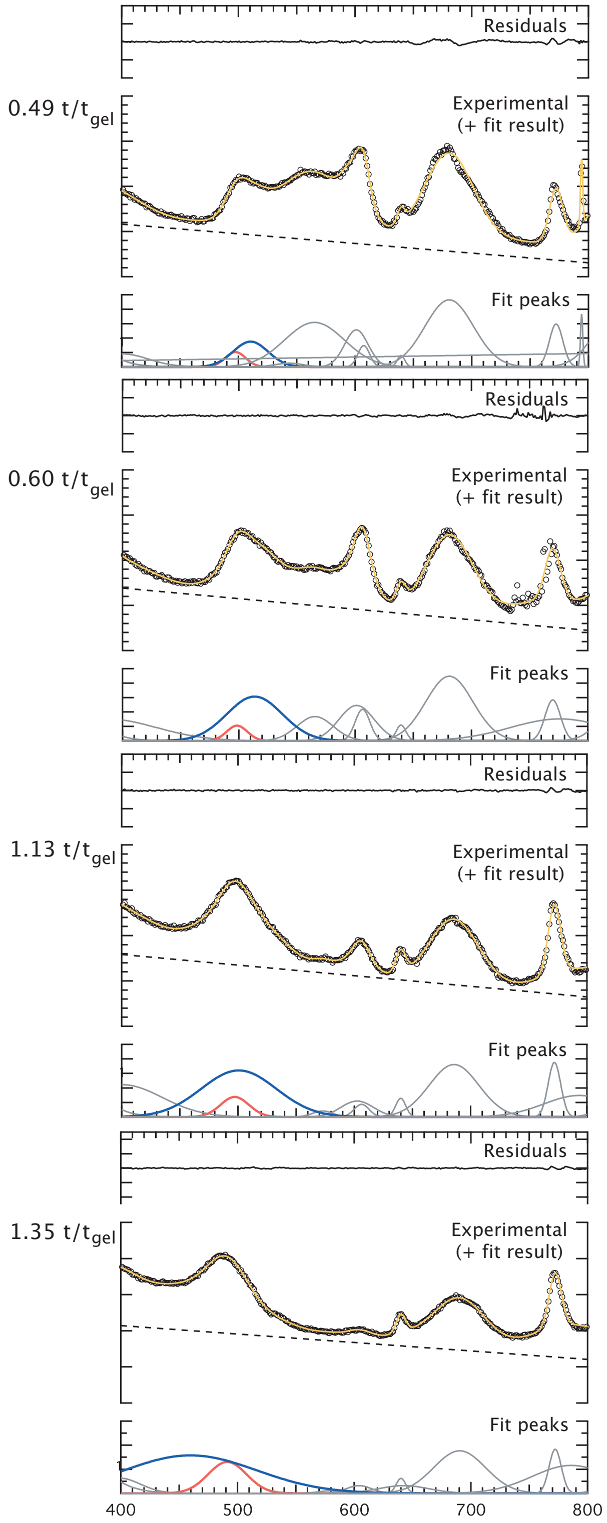

Figure S2. Raman spectra recorded for the aged gel (black) and the aged ionogel (red), *i.e.*, after 7 months from preparation. For the ionogel, the spectrum shown is indeed a difference spectrum between the ionogel and the pure ionic liquid, to reveal the spectral features due to silica.

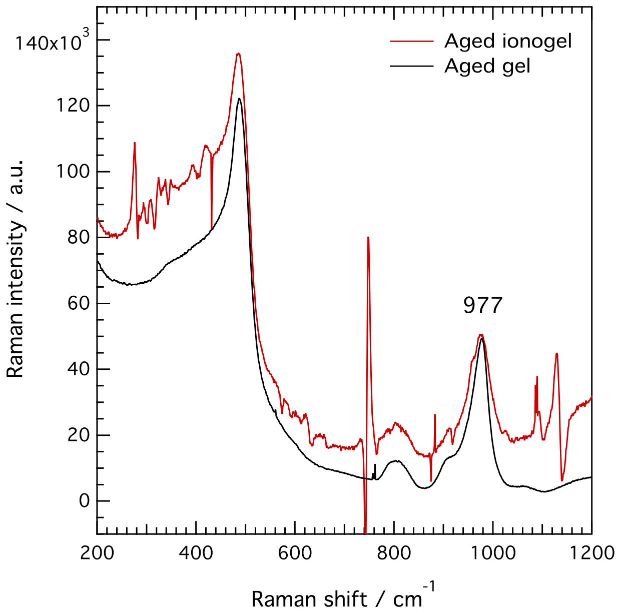

© 2014 by the authors; licensee MDPI, Basel, Switzerland. This article is an open access article distributed under the terms and conditions of the Creative Commons Attribution license (http://creativecommons.org/licenses/by/3.0/).
